# Context familiarity speeds up boundary responses at encoding

**DOI:** 10.1007/s00426-026-02316-3

**Published:** 2026-05-26

**Authors:** Vincent van de Ven, Noga Baake, Marianna Kardas, Aaron Krater, Gustė Michelevičiūtė

**Affiliations:** Faculty of Psychology and Neuroscience, Department of Cognitive Neuroscience, Maastricht University, Maastricht, Netherlands

## Abstract

In event segmentation, a context shift (event boundary) commonly leads to slower response times, compared to response times for actions occurring away from boundaries. This effect is commonly considered to result from novelty and segmentation processes in working memory. It is less clear if and how familiarity affects boundary processing. In the current study, we investigated the role of contextual familiarity in boundary processing. Participants saw lists of random pictures while simultaneously listening to sound contexts that changed after each triplet of pictures and rated for each picture how much they thought it was associated to the context. Contextual familiarity was manipulated by replaying one of the sound contexts three additional times throughout a list. During list presentation, participants showed increasingly faster boundary response times for each repetition of the sound context, compared to novel contexts. This effect was not due to merely playing more contexts over time. Participants showed no boundary-related effect in the associative ratings. A subsequent temporal order memory task showed no effect of context familiarity on memory performance, suggesting boundary-related processing and event memory formation may depend on partly independent processes.

## Introduction

We learn from our environment by detecting and responding to changes. The ability to recognize shifts in context allows us to update our mental representations, adapt to new circumstances, and anticipate future events. These contextual changes define event boundaries that separate one event from another and include notable shifts in place or time, a change in the social environment or different action goals. According to event segmentation theories, the brain identifies these boundaries by detecting discrepancies between expected and actual sensory input, resulting in a mismatch representation or prediction error, which signals when the current event model in working memory is no longer sufficient and must be updated (Brunec et al., 2018; Radvansky & Zacks, 2017; Zacks, 2020). A common behavioral finding is that participants take longer to respond to items presented at a contextual change (boundary items), compared to items further away from those changes (non-boundary items). For example, when reading narrative texts, participants’ reading times were significantly longer for words that indicated a shift in context or perspective (Pettijohn & Radvansky, 2016; Rinck & Weber, 2003; Speer & Zacks, 2005; Swets & Kurby, 2016; Zwaan et al., 1998). A similar effect can be observed when participants cross (virtual) rooms, extending the boundary effect to spatial context (Radvansky & Copeland, 2006). A comparable effect has also been found in studies in which participants saw lists of random pictures that were presented in perceptually changing contexts, such as the color of the frame around the picture or an accompanying image of a scene. Contextual features changed after several consecutively presented items, thereby mimicking event boundaries in naturalistic stimuli. During the presentation of the picture list, participants had to make a judgment about the relation between each item and its context. Results showed slower response times for pictures presented right after a contextual feature change (Heusser et al., 2018; van de Ven et al., 2023; Wang et al., 2024; Xiang et al., 2025), in line with the boundary effect of reading times of narrative texts. The slower boundary responses could reflect increased attentional demands during event model updating in working memory (Ongchoco & Scholl, 2019; Pradhan & Kumar, 2022), during which participants are less able to detect visual targets at boundaries, compared to target detection further away from boundaries (Huff et al., 2012; Pradhan & Kumar, 2022) or retrieve prior information from memory (Speer & Zacks, 2005). Further, Heusser et al. (2018) reported that relatively slower boundary responses predicted worse temporal order memory judgments for items that were presented on either side of those boundary items, compared to faster boundary responses, supporting the notion that boundary response times are related to boundary processing.

However, slower boundary responses may also reflect other processes co-occurring with but mechanistically unrelated to context changes. For example, response priming to a new context could speed up responses for subsequent items after having responded to the boundary item (Chun & Jiang, 1998; Henson et al., 2014). After establishing a response choice for the first item, that specific choice or a subset of related choices could then be easily reactivated for subsequent items while that context remains stable, resulting in faster responses for subsequent items, until a new context is presented. Such processes may correlate with serial position within a context, and even be triggered by a context change, but would not contribute to boundary processing and segmentation. Most previous studies did not investigate alternative explanatory scenarios underlying responses to boundaries.

While much research has focused on the role of perceptual novelty in segmentation, less is known about how the familiarity of a context might influence boundary processing. Familiarity with a context could influence boundary formation in multiple ways. A more familiar context could provide a richer and more stable cognitive framework, for which individuals may be more tolerant of minor deviations, leading to a reduced likelihood of forming event boundaries (Michelmann et al., 2021; Schoenenkorb et al., 2023). Familiarity might also affect the meaningfulness of a context shift (Bläsing, 2014; Newberry & Bailey, 2019), thereby enhancing the saliency of that shift.

The interplay between novelty and familiarity also has important implications for event memory (Quent et al., 2021; Van Kesteren et al., 2012). Prior research has shown that event boundaries serve as anchors in episodic memory, improving recall of information that occurs at these junctures (Ben-Yakov & Dudai, 2011; Silva et al., 2019). Novelty is widely associated with enhanced neural plasticity and improved memory (Bunzeck & Düzel, 2006; Duszkiewicz et al., 2019; Lisman & Grace, 2005). However, the role of familiarity in memory formation is complex. Familiarity and novelty are processed in partially distinct neural systems and interact with expectation and task demands (Kafkas & Montaldi, 2018; Quent et al., 2021). Familiarity in terms of prior knowledge or pre-existing schemas scaffold encoding and improve consolidation for new related information (Bower et al., 1979; Gilboa & Marlatte, 2017; Newberry & Bailey, 2019; Van Kesteren et al., 2012). Further, experimentally familiar items can be remembered better than novel ones (Poppenk et al., 2010). Conversely, increasing familiarity via simple repetition can lead to reduced neural responses and reduced attention to stimulus details (Grill-Spector et al., 2006; Yi & Chun, 2005). Repetition and prior knowledge may even lead to comparable neural suppression effects (Poppenk et al., 2016). However, the effect of repetition on neural processing depends on expectation, predictability and task demands (Auksztulewicz et al., 2017; Kafkas & Montaldi, 2018; Summerfield & Egner, 2009). Expected repetitions tend to produce repetition suppression, whereas unexpected repetitions produce larger prediction errors that can drive stronger encoding and engage hippocampal pattern separation mechanisms that benefit memory for contextual detail. In short, familiarity can both enhance and attenuate memory formation, depending on other cognitive and task-related factors.

In the current study, we aimed to investigate how familiarity with a context modulates event segmentation. We propose that familiar contexts reduce the cost of event model updating in working memory, leading to faster boundary responses compared to novel contexts. To test this suggestion, we had participants view lists of randomly selected objects while listening to sound contexts and make a judgment about the relation between each object and the accompanying sound context. Sound contexts changed after every third visual object, thereby inserting event boundaries. In addition, for each list, one of the auditory contexts would be repeated over the course of the list and increase the familiarity with that context. Participants did not know up front which context would be repeated nor could they predict when a repeated context would be presented. We hypothesized that response times for boundary items at the start of a repeated context would diminish with subsequent context repetitions, compared to boundary responses for novel contexts, and irrespective of the relational judgments. To test if contextual familiarity influenced event memory, we assessed performance in a temporal order memory task. Accuracy for temporal order judgments is commonly higher when the tested items shared the same event context at encoding, compared to items that were separated by an event boundary (DuBrow & Davachi, 2016; Heusser et al., 2018; Pu et al., 2022; van de Ven et al., 2023), due to boundaries interfering with associative temporal encoding (Heusser et al., 2018; Pu et al., 2022). Further, slower boundary response times may predict worse temporal order memory performance for test items crossing boundaries (Heusser et al., 2018). Based on this finding, we hypothesized that if increased familiarity diminished boundary processing in terms of faster response times, then across-events temporal order accuracy would improve, compared to boundaries related to novel contexts.

We conducted two experiments with equal statistical power with the following aims. First, we wanted to replicate the effect of familiarity of Experiment 1 with an independent sample. Second, we used different contextual sounds, with only neutral sounds in Experiment 1 and negative emotional contexts in Experiment 2, based on previous experiments showing stronger boundary-related processing for emotional contexts compared to neutral contexts (McClay et al., 2023; Wang & Lapate, 2025). We expected that the effect of context familiarity in Experiment 1 would be replicated more strongly in Experiment 2.

## Participants

Between both experiments, we recruited a total of 62 participants from the student cohort of the Faculty of Psychology and Neuroscience (Experiment 1: *N* = 31, mean (SD) age = 20.8 (2.11) years; 24 self-reported females and 7 males; Experiment 2: *N* = 31, mean (SD) age = 22.6 (3.89) years; 28 self-reported females and 3 males). There were no overlapping participants between the two experiments. Participants received course credit for their participation. We estimated sample size for an experiment using G*Power (Faul et al., 2007) based on a two-tailed paired samples t-test with significance level set to 0.05, statistical power of 0.8 and an effect size of 0.6 (see also (DuBrow & Davachi, 2013; van de Ven et al., 2023)), yielding a minimum sample size of 24. All participants provided written informed consent prior to participation. The experiment was approved by the ethics committee of the Faculty of Psychology and Neuroscience of Maastricht University.

## Procedures

### Visual and auditory stimuli

Images were taken from a publicly available data set displaying random objects (Kovalenko et al., 2012). Images were preselected to exclude those that could potentially induce emotional valence. Images were converted to grayscale and displayed on a gray background (300 × 300 dpi). During each experiment, images were presented in a randomized order for each participant.

For Experiment 1, we selected 48 non-emotional, neutral sound stimuli from the International Affective Digitized Sounds – Emotional database (IADS-E, https://sites.google.com/view/iads-e/; Yang et al., 2018) as auditory contexts. Of each sound stimulus, the first 3 s (of the full duration of 6 s) were played during the encoding phase of the experiment (see below). For Experiment 2, we selected 24 neutral and 24 negatively valenced sound stimuli. The sounds in this database were previously rated for emotional valence and arousal using a 9-point Likert scale that ranged from very positive (1) to very negative (9). We selected neutral stimuli based on these valence ratings between 5.00 and 6.50 and negatively valenced stimuli with ratings above 6.50. Sounds were presented through headphones.

### Experimental design

Each experiment was performed on a Windows-controlled PC in a dedicated lab for behavioral testing. Participants were seated in front of a PC monitor and were required to wear headphones (Corsair Model RDA 0011) at the start of the experiment. They then received instructions for the completion of an encoding task and a temporal order memory task. The experiment was programmed using PsychoPy (Bridges et al., 2020).

**Fig. 1:**
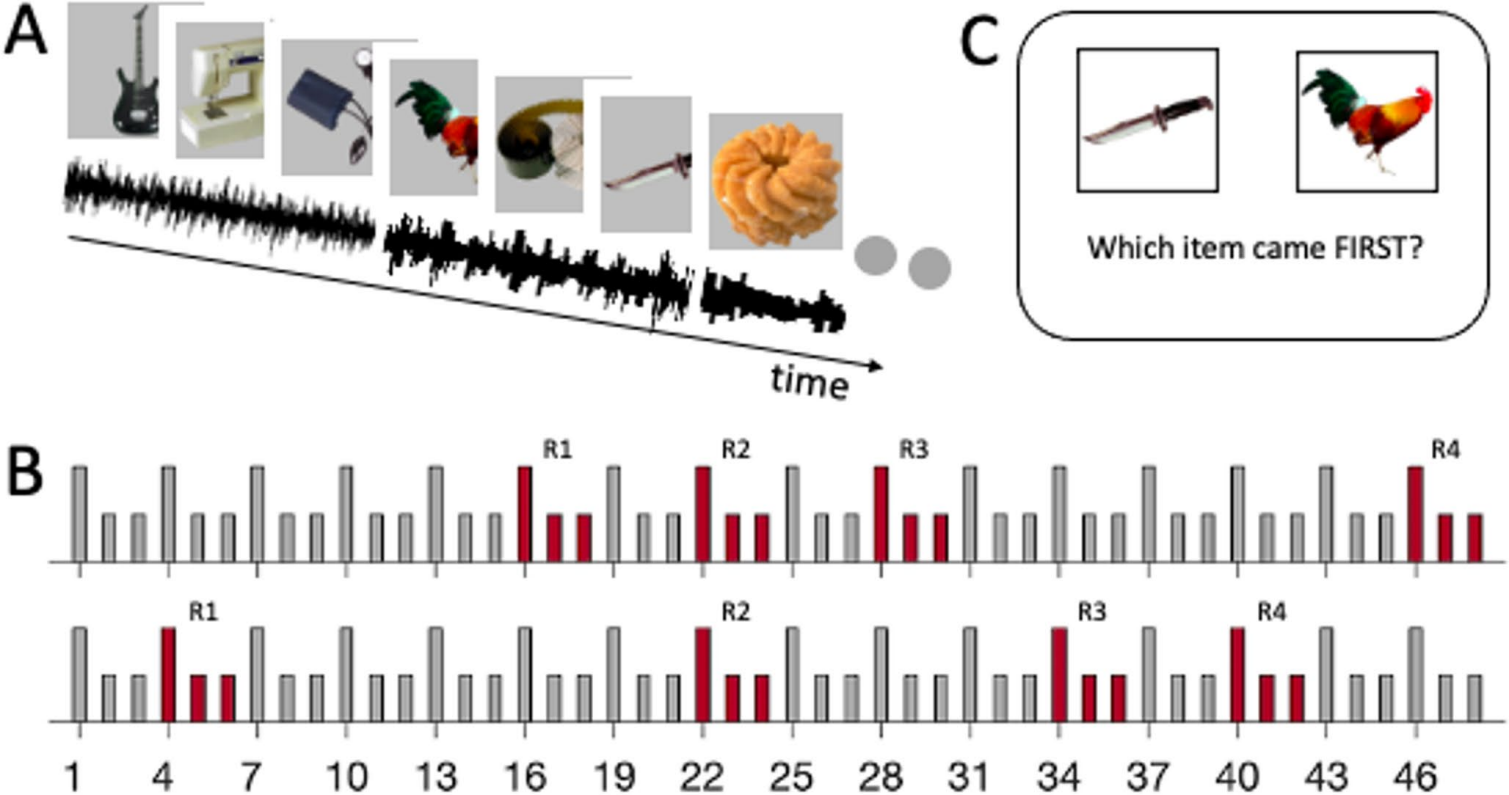
Experimental design. **A**: Pictures and sounds were presented simultaneously, with a sound playing across three consecutive pictures (triplets). **B**: Two examples of picture triplets with novel sounds (grey) and the repeated sound (red bars, R1 – R4 indicate repetitions). Higher bars indicate boundary items of a triplet, at which a new sound stimulus started. Pictures were presented in grayscale during the experiment. One of the sounds in a list was played over four triplets at pseud-random moments, increasing familiarity with that sound as the list progressed. **C**: After each list, participants performed a temporal order memory test of the previously presented pictures

Each participant in both experiments completed four runs of an encoding and a temporal memory task. Stimuli were not repeated across runs. In the encoding task of each run of each experiment (Fig. 1A), participants were presented with a series of 48 pictures and accompanying sound stimuli as auditory contexts. Each picture was presented for 3 s and was followed by an inter-stimulus-interval of 2 s before the next picture was presented. During each picture presentation, participants heard a sound stimulus that was played for 3 s from start to cessation of picture presentation and had to rate how much they thought the picture was associated to the co-presented sound on a 4-point Likert Scale with 1 indicating “very much” and 4 “not at all”. There was a maximum response window of four seconds for each picture/sound pair. A sound stimulus was repeated for three consecutive picture stimuli (triplets), after which a new sound stimulus was chosen. Further, in each round of the encoding task, one sound stimulus was selected to be repeated three more times during the series of pictures, such that familiarity with that sound stimulus increased compared to the other, non-repeated sound stimuli (Fig. 1B). Thus, the presented sounds included twelve once-presented sounds (Non-familiar) and one sound stimulus that was presented four times (Familiar). Repetitions of the selected sound stimulus were separated in time by at least one novel sound. In Experiment 1, all sound stimuli were of neutral valence. In Experiment 2, every second sound stimulus was negatively valenced, such that neutral and emotional sounds alternated throughout the encoding task. In a post-segmentation rating task after completing the four runs of Experiment 2, participants rated the negative contexts as significantly more negative in emotional valence than neutral contexts (t(30) = 32.32, 95% CI=[1.80, 2.05], p = < 0.001, Cohen’s d = 5.81), indicating that they experienced the emotional impact of the contexts in accordance with our selection of neutral and negative contexts.

After the encoding task, participants completed a temporal order memory task comprising thirty trials, in which each trial contained two pictures selected from the previously seen picture series. Half the picture pairs were selected from the same sound context (Within-event) and half from different sound contexts (Across-events). The items of each pair were separated by one additional picture in the previously presented series. Participants had to indicate which of the two stimuli was presented first during the encoding task. Response options included “Sure LEFT”, “Probably LEFT”, “Probably RIGHT” and “Surely RIGHT”. There was no response time limit. In total, participants completed 120 trials across all four runs. For data analysis, we dichotimized the responses by grouping the Sure and Probably Left responses as „Left“ and the Sure and Probably Right responses as „Right“ responses, which were then compared against the left or right item truly having been presented first in the series.

## Results

### Encoding response times of non-familiar and familiar contexts

#### Experiment 1

**Fig. 2:**
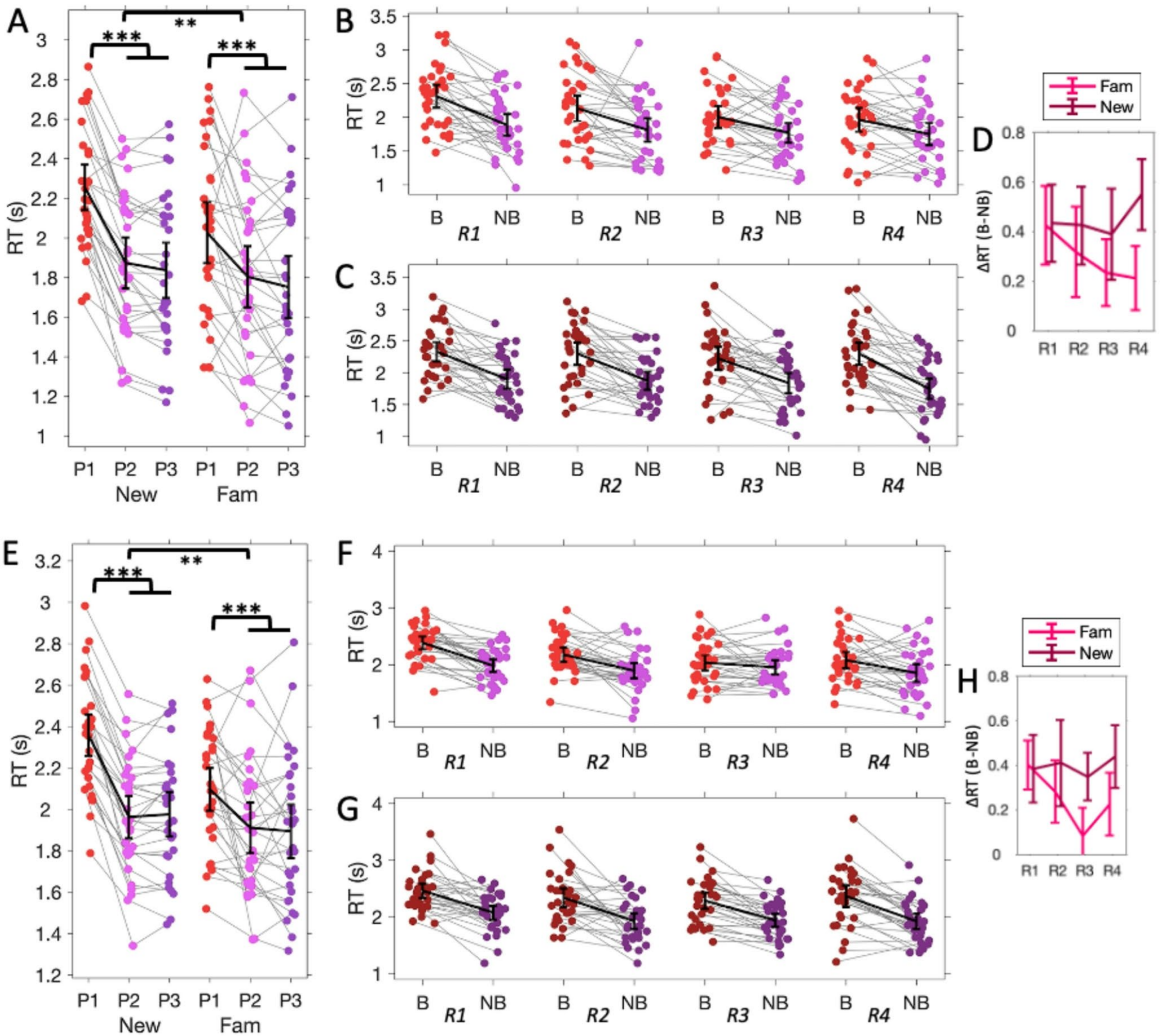
Segmentation response time results. Encoding response time results for Experiment 1 (**A**-**D**) and 2 (**E**-**H**). **A**, **E**: Response times for each the three positions of novel and familiar contexts. **B**, **F**: Encoding response times for the familiar contexts for the boundary (position P1 in red) and non-boundary positions (pooling positions 2 [pink] and 3 [purple]) for each repetition (R1 – R4) of the familiar sound. **C**, **G**: Encoding response times for novel contexts sampled across comparable positions within the series. **D**, **H**: Response time difference (ΔRT) between boundary and non-boundary responses for the repeated familiar (pink) and new contexts (purple). Error bars indicate 95% confidence interval. *** *p* < 0.001

Figure 2A shows the distribution of encoding response times across participants for each of the three positions within a sound context. In the following analyses, we compared the Boundary position (position 1 of an auditory context) with Non-boundary positions (pooling positions 2 and 3 of an auditory context). To test if encoding response times varied with familiar vs. unfamiliar sound boundaries, we conducted a repeated measures ANOVA with Picture Position (Boundary vs. Non-Boundary positions) and Sound Familiarity (unfamiliar vs. familiar sound) as within-subject factors. The ANOVA revealed a significant effect of Position (F(1,30) = 69.95, *p* < 0.001, $\:{\eta\:}_{p}^{2}$=0.70), a significant effect of Familiarity (F(1,30) = 36.68, *p* < 0.001, $\:{\eta\:}_{p}^{2}$=0.55) and a significant Position ⋅ Familiarity interaction effect (F(1,30) = 8.61, *p* = 0.006, $\:{\eta\:}_{p}^{2}$=0.22). These results indicated that the boundary responses were faster for the familiar than the novel contexts. A posthoc comparison between the boundary responses of the novel and familiar contexts statistically supported this finding (T(30)=-5.48, 95% CI=[-0.14, -0.31], *p* < 0.001, Cohen’s d=-0.98).

**Fig. 3:**
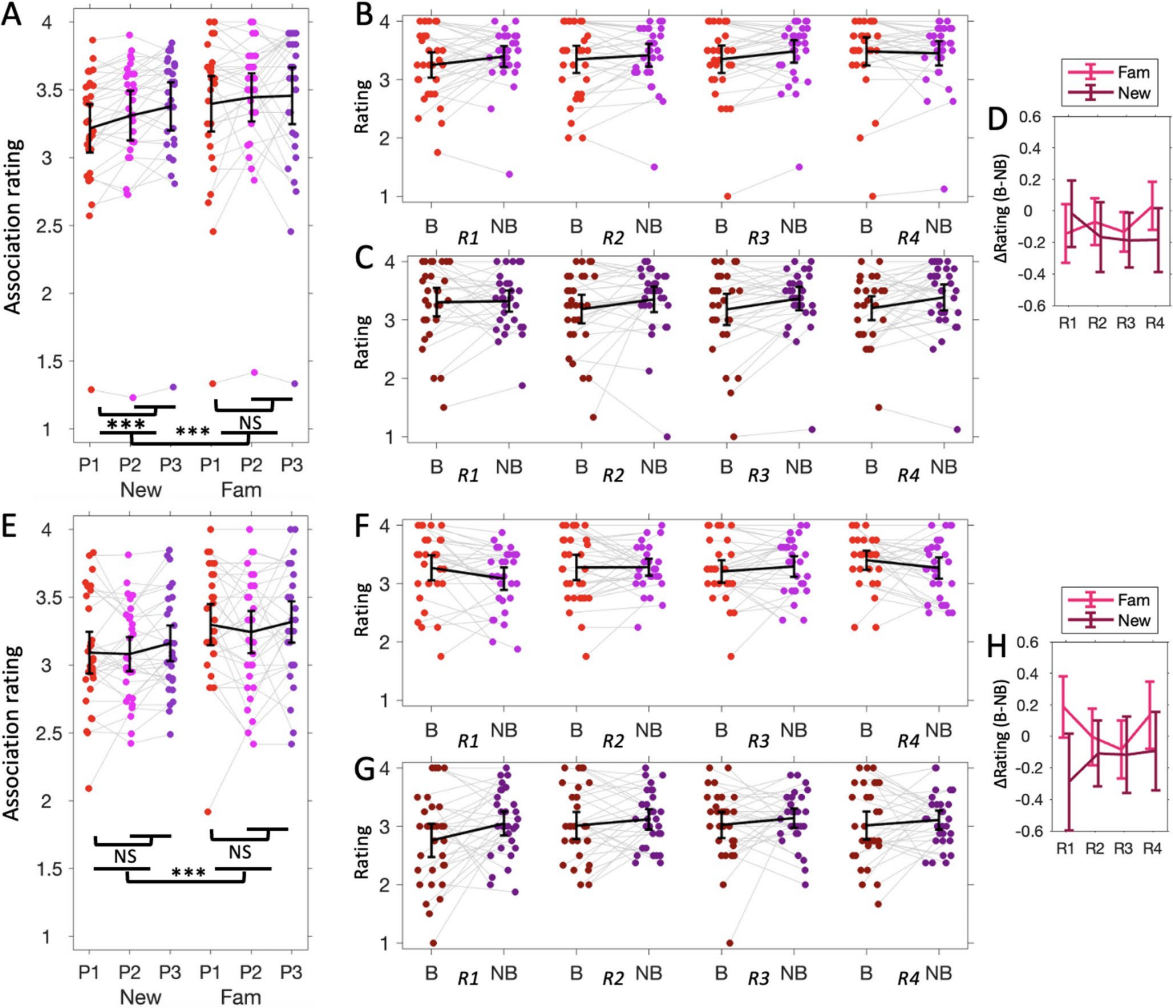
Segmentation rating results. Associative rating results for Experiment 1 (**A**-**D**) and 2 (**E**-**H**). Panels and legends same as in Fig. 2. Error bars indicate 95% confidence interval. *** *p* < 0.001, NS not significant

To further investigate how context repetition affected boundary response times for the familiar context, we tested if the response time difference between the boundary and non-boundary items linearly decreased with context repetition (Fig. 3B). We found that the linear contrast was statistically significant (T(30) = 2.32, 95% CI=[0.04, 0.68], *p* = 0.027, Cohen’s d = 0.42).

To test if the linear effect did not simply arise from presenting contexts over time, we sampled response times for the novel contexts that immediately preceded the repeated contexts (Fig. 3C). The linear contrast was not significant (T(30)=-1.01, *p* = 0.32). To evaluate the evidence of no difference, we calculated the Bayes Factor (BF10) equivalent of the T-test using the *Bayes Factor toolbox* for Matlab, which uses a Cauchy prior with a scale of 0.707 (Rouder et al., 2012). We found moderate evidence for no difference (BF10 = 0.31; in accordance with suggested evidence categorization for BF10 < 0.33, Schönbrodt & Wagenmakers, 2018). Comparing the linear contrasts for the repeated and novel contexts showed a significant effect (T(30)=-2.52, *p* = 0.017, Cohen’s d=-0.45; Fig. 3D), statistically verifying that boundary response times decreased with subsequent repetition of a context, rather than the passage of time.

#### Experiment 2

We first assessed if boundary responses differed between neutral and negative contexts (excluding the repeated contexts) using a paired samples T-test, which showed no significant difference (T(30)=-1.01, *p* = 0.32). The Bayes Factor indicated moderate evidence for no difference between the two contexts (BF10 = 0.31). We therefore pooled the response times of the novel neutral and negative conditions. We then used a repeated measures ANOVA that was comparable to that of Experiment 1, with Picture Position (Boundary vs. Non-Boundary positions) and Sound Familiarity (unfamiliar vs. familiar sound) as within-subject factors. Results again showed a significant effect of Position (F(1,30) = 71.27, *p* < 0.001, $\:{\eta\:}_{p}^{2}$=0.70), a significant effect of Familiarity (F(1,30) = 27.60, *p* < 0.001, $\:{\eta\:}_{p}^{2}$=0.48) and a significant Position × Familiarity interaction effect (F(1,30) = 22.68, *p* < 0.001, $\:{\eta\:}_{p}^{2}$=0.43). Boundary responses were slower than non-boundary responses, but this difference was smaller for the familiar contexts than for the novel contexts (Fig. 3E). We conducted linear contrasts of the decreasing difference between the boundary and non-boundary responses for the repetitions of the familiar context and for novel contexts presented at comparable intervals as the repetitions (Fig. 3F-H). The linear contrast was significant for the familiar context (T(30) = 3.15, *p* = 0.004, Cohen’s d = 0.57) but not the novel contexts at comparable intervals (T(30)=-0.31, *p* = 0.76), of which the Bayes Factor indicated moderate evidence of no difference (BF10=0.20).

### Encoding ratings of non-familiar and familiar contexts

Figure 3 shows the distribution of associative ratings for each of the three positions within a sound context for each of the two experiments. Overall, participants generally indicated that they thought that the pictures were not strongly associated to the auditory contexts (Experiment 1 mean = 3.35, 95% CI=[3.17, 3.53]; Experiment 2 mean = 3.20, 95% CI=[3.07, 3.23]). To test if familiar contexts affected boundary vs. non-boundary ratings, we used a repeated measures ANOVA model with Position (Boundary vs. Non-boundary positions) and Familiarity as within-subject factors. For Experiment 1 (Fig. 3A-D), results showed a significant main effect of Position (F(1,30) = 13.79, *p* < 0.001, $\:{\eta\:}_{p}^{2}$=0.31) and Familiarity (F(1,30) = 24.30, *p* < 0.001, $\:{\eta\:}_{p}^{2}$=0.45), but no significant Position × Familiarity interaction effect (F(1,30) = 3.11, *p* = 0.088, $\:{\eta\:}_{p}^{2}$=0.09). Posthoc comparisons showed a boundary effect for novel (T(30) = 5.03, *p* < 0.001, Cohen’s d = 0.90) but not for familiar contexts (T(30) = 1.40, *p* = 0.17, Cohen’s d = 0.25), although the ANOVA interaction effect was not significant. Further, the relative boundary to non-boundary ratings did not change with repetition of the familiar (T(30)=-1.25, *p* = 0.22, Cohen’s d=-0.22, BF10=0.39; Fig. 3B) or the novel contexts (T(30) = 1.28, *p* = 0.21, Cohen’s d = 0.23, BF10=0.40; Fig. 3C).

Similar results were obtained from Experiment 2 (Fig. 3E-H). The repeated measures ANOVA yielded a significant effect of Familiarity (F(1,30) = 18.30, *p* < 0.001, $\:{\eta\:}_{p}^{2}$=0.38), but not for Position (F(1,30) = 0.03, *p* = 0.87, $\:{\eta\:}_{p}^{2}$=0.0) or the interaction term (F(1,30) = 0.37, *p* = 0.55, $\:{\eta\:}_{p}^{2}$=0.01). Further, the relative boundary to non-boundary ratings did not change with repetition of the familiar (T(30) = 0.53, *p* = 0.60, Cohen’s d = 0.10, BF10=0.22; Fig. 3F) or novel contexts (T(30)=-1.20, *p* = 0.24, Cohen’s d=-0.22, BF10=0.37; Fig. 3G). These findings suggest that, in contrast to response times, the ratings did not vary with subsequent repetition of the familiar contexts.

### Temporal order memory

**Fig. 4:**
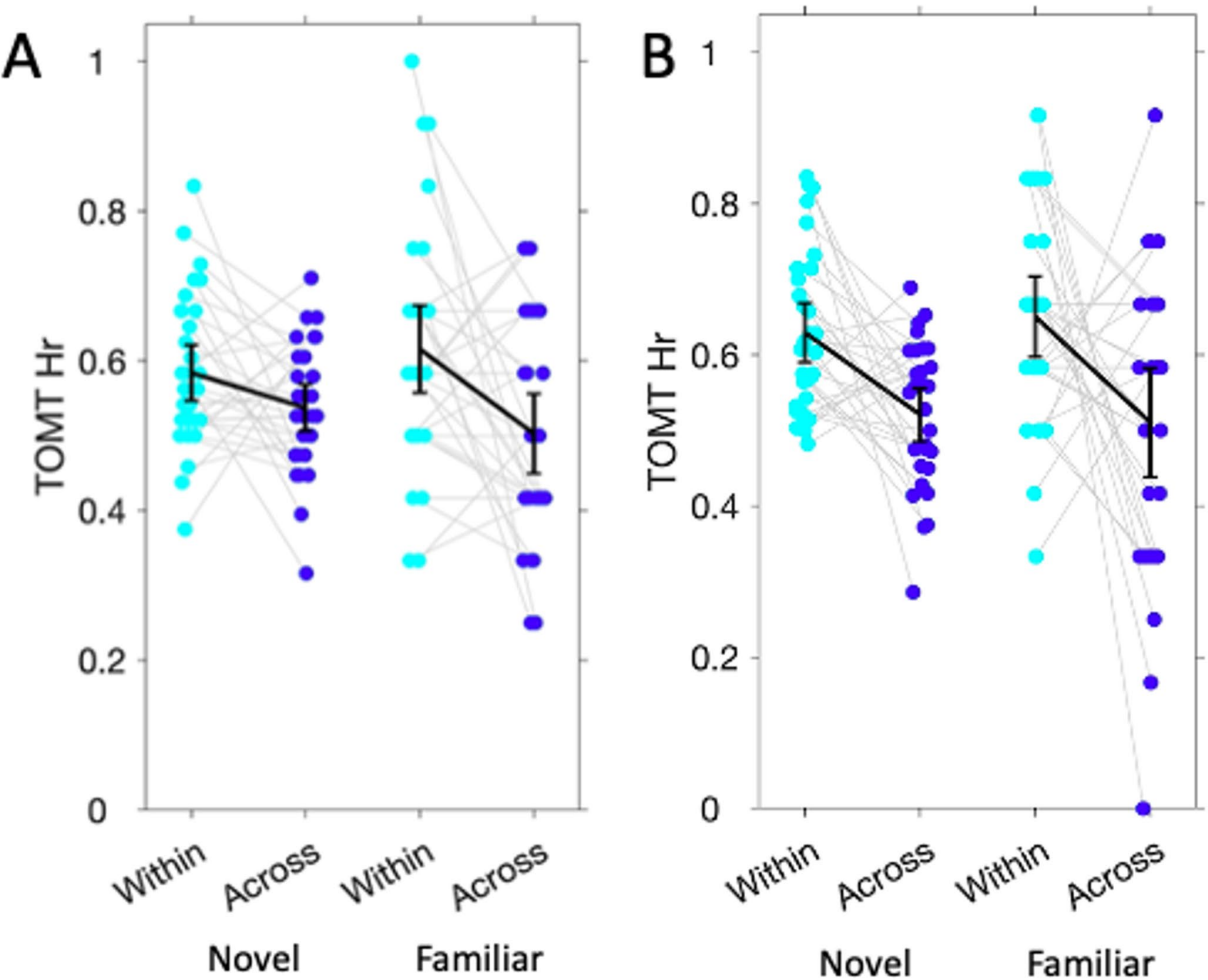
Temporal memory results. Temporal order memory hit rates for the within and across event pairs for pictures drawn from the novel and familiar conditions for Experiment 1 (**A**) and 2 (**B**). Error bars indicate 95% confidence interval.

Table 1 and Figure 4 show the temporal order memory hit rates for each of the four conditions. We analysed hit rates using a repeated measures ANOVA with Context (within-event, across-events) and Familiarity as within-subject factors for each experiment separately.

For Experiment 1, we found a significant main effect of Context, F(1,30) = 10.48, *p* = 0.003, $\:{\eta\:}_{p}^{2}$=0.26, with higher hit rates for the Within-event pairs compared to the Across-events pairs. We found no significant effect of Familiarity, F(1,30) = 0.01, *p* = 0.94, $\:{\eta\:}_{p}^{2}$=0.0, and no significant Context × Familiarity interaction, F(1,30) = 2.43, *p* = 0.13, $\:{\eta\:}_{p}^{2}$=0.07.

For Experiment 2, we first tested if Within-context hit rates differed between the neutral and negative contexts and found no significant difference (T(30) = 0.82, *p* = 0.42). The BF10 for this comparison was 0.26, indicating moderate evidence for no difference of contextual emotional valence. We therefore pooled the hit rates of the novel neutral and negative contexts. A repeated measures ANOVA with Context (within-event, across-events) and Familiarity as within-subject factors revealed a significant main effect of Context (F(1,30) = 10.80, *p* = 0.003, $\:{\eta\:}_{p}^{2}$=0.26), but no significant effect of Familiarity (F(1,30) = 0.11, *p* = 0.75) and no significant Context × Familiarity interaction (F(1,30) = 0.56, *p* = 0.46). Thus, we again found no evidence that context familiarity would affect temporal order memory performance, compared to novel contexts. In sum, both experiments showed that familiarity with a sound context did not affect temporal order memory performance, compared to novel contexts, regardless of emotional valence.

**Table 1:** Temporal order memory results. Hit rate statistics for each condition of the temporal order memory task for Experiments 1 and 2. One sample T-tests were calculated against chance performance of 0.5. Degrees of freedom in each experiment was 30. * significant after Bonferroni correction (per Experiment)

Exp	Context	Boundary	M (SE)	T(30)	*P*	Cohen’s d
1	New	Within	0.58 (0.02)	4.60	< 0.001*	0.83
		Across	0.54 (0.02)	2.44	0.021	0.44
	Familiar	Within	0.62 (0.03)	4.05	< 0.001*	0.73
		Across	0.50 (0.03)	0.10	0.92	0.02
2	New	Within	0.63 (0.02)	6.76	< 0.001*	1.21
		Across	0.52 (0.02)	1.21	0.24	0.22
	Familiar	Within	0.65 (0.03)	5.78	< 0.001*	1.04
		Across	0.51 (0.03)	0.30	0.76	0.05

## Discussion

In two experiments, we manipulated the familiarity of contextual sounds to test its effect on boundary processing and performance in a subsequent temporal memory task. We replicated previous reports of slower responses for boundary items that were presented at a contextual change, compared to non-boundary items presented further away from those changes (Heusser et al., 2018; van de Ven et al., 2022; Ven et al., 2023; Wang et al., 2024; Xiang et al., 2025). However, we also found that boundary response times decreased for more familiar contexts that were repeated throughout a list. More specifically, boundary response times linearly decreased with subsequent repetitions of the selected context, but not with the mere passage of time, suggesting an incremental effect of increasing familiarity of the repeated context on the processing of an event boundary.

The ratings for picture-sound associations did not differ between boundary and non-boundary items during encoding. Taken together with the response time findings, participants responded slower for the same rating choices for boundary items compared to non-boundary items. Participants did rate pictures as less associated to familiar than to novel contexts, which aligns with reports that familiarity increases stimulus salience (Jacobsen et al., 2005; Moldakarimov et al., 2010) and reduces the likelihood of forming cross-stimulus associations, compared to unfamiliar or novel stimuli (Baumann et al., 2020).

Contrary to our hypothesis, we found no evidence that context familiarity affected temporal order memory. That is, participants showed the typical superiority of within-event over across-events temporal order accuracy (Heusser et al., 2018; Pu et al., 2022; van de Ven et al., 2023) for both novel and familiar contexts. This suggests that the increasing familiarity of a context did not decrease or interfere with the temporal associative memory formation of the pictures presented with that context. Thus, boundary-based memory formation seems to depend on the presence of a contextual change, rather than what a context changes to.

Taken together, our findings seem consistent with a context priming account, rather than with a pure boundary processing account or a prediction error account. From the perspective of a boundary processing account, a contextual change taxes event model updating in working memory, such as by increasing attentional resources to process the change or by flushing the previous event model from working memory to form a new event model that better captures the current experience (Güler et al., 2024; Ongchoco & Scholl, 2019). These processes delay subsequent cognitive operations during perception, thereby leading to slower boundary responses. Context repetition would make memory updating less demanding as a previously formed event model can be reactivated more easily and thus require fewer attentional resources during event model updating. This would lead to faster boundary response times, which we observed, but also enhanced temporal memory accuracy (cf. Heusser et al., 2018), which we did not observe. A prediction error account (Zacks, 2020; Zacks et al., 2011) faces a similar problem: While reduced prediction error at familiar context onsets could explain faster boundary response times, it would also reduce encoding of boundary items and alter the structure of event memory, which was not observed. Instead, context priming (Bainbridge et al., 1993; Chun & Jiang, 1998) can arguably best explain the speeding up of boundary responses with context repetition without affecting temporal memory accuracy. A novel context introduces a response cost at context onset, but subsequent responses in that context speed up as the contextual features prime the cognitive or response set for subsequent items. When that context is later shown again, the contextual features stored in memory reinstate the cognitive set to facilitate responding, with a larger relative advantage for the boundary item than for subsequent items. This further aligns with our finding that ratings did not vary across position within a context, despite items being different from each other. Finally, context priming may not influence temporal memory formation of the segmented events. Notably, this view contrasts a previous finding of slower boundary response times predicting decreased temporal order memory performance, compared to relatively faster boundary response times (Heusser et al., 2018), suggesting that boundary response times are indicative of boundary processing and segmentation. In that study as well as ours, participants were asked to make a judgment about the relation between item and context, although the exact instructions varied slightly between the two studies. More generally, it remains unclear if this effect in picture list paradigms such as ours is related to response slowing at event boundaries in text reading or other naturalistic settings (Pettijohn & Radvansky, 2016; Radvansky & Copeland, 2006; Zwaan et al., 1998). Future studies should investigate which cognitive factors influence boundary response times and subsequent memory performance.

Finally, we found that the inclusion of emotional contexts did not significantly change temporal memory performance. The rating task in Experiment 2 confirmed that participants did experience the affective contexts as more emotionally negative than the neutral contexts. Several previous studies found that negative emotional contexts enhanced subsequent memory for temporal order, possibly due to emotional valence or arousal facilitating attentional encoding and memory formation (Riegel et al., 2025; Wang & Lapate, 2025). However, other studies did not report enhanced temporal order memory from negative emotional contexts (Ceccato et al., 2022; McClay et al., 2023), suggesting that other factors may play a role in how emotions affect event memory formation.

More generally, our findings fit with results from other studies using multi-sensory segmentation, in which context changes of one sensory modality affect event memory formation of items in another modality. In a study by Clewett and McClay (2025), participants watched series of pictures and heard an emotional or neutral sound clip at regular intervals within a series, which acted as an auditory event boundary. Response times were slower for boundary than for non-boundary items, regardless of the valence of the boundary clip. Another study used multi-sensory audio-visual contexts of frame color and soundscapes to segment picture lists and found slower boundary response times for auditory and audio-visual boundaries than for visual boundaries, perhaps due to the higher salience of the soundscapes than the frame colors (van de Ven et al., 2023). Both studies also reported worse temporal order memory performance for visual items crossing auditory boundaries, with poorer performance when crossing synchronous (multi-sensory) as opposed to asynchronous (uni-sensory) boundaries (van de Ven et al., 2023) or for more arousing auditory boundaries (Clewett & McClay, 2025). McClay et al. (McClay et al., 2023) had participants watch a series of pictures while listening to songs in which perceptual and emotional features varied throughout each song. Subsequent temporal order memory performance showed poorer performance for picture pairs crossing perceptual as well as emotional valence (but not arousal) boundaries, compared to pictures drawn from song segments without such boundaries. In all, these and our studies show cross-modality segmentation in which context boundaries in one sensory modality affect segmentation of content in another modality.

A limitation of our study is that we did not use other measures to assess how repeated contexts influenced segmentation and memory formation. For example, boundaries can enhance encoding of object-context associations (Heusser et al., 2018; van de Ven et al., 2022). It is possible that repeated contexts may affect object-context associations, as items presented at very different positions within a list become bound to the same context, thereby leading to interference or confusion. Another limitation is there were fewer memory trials available for the familiar, compared to the novel conditions, which could have obfuscated a potentially small effect of context familiarity on temporal memory accuracy. We suggest that these limitations should be addressed in future studies.

In conclusion, we showed that increasing familiarity through context repetition leads to faster boundary responses during encoding. This effect was not related to the passage of time or temporal prediction of when a familiar context would be presented. Context familiarity did not alter boundary-based temporal order memory performance, suggesting that the presence of a context change, rather than what a context changes to, is relevant for event memory formation.

## Data Availability

Behavioral data are publicly available on the Open Science Foundation page. Available at 10.17605/OSF.IO/SQPJA.

## Declarations

### Generative AI and AI-assisted technologies in the writing process

Generative AI was used to improve textual flow and readability.
